# Long non-coding RNA MALAT1 contributes to cell apoptosis by sponging miR-124 in Parkinson disease

**DOI:** 10.1186/s13578-017-0147-5

**Published:** 2017-04-21

**Authors:** Wei Liu, Qishun Zhang, Jianlei Zhang, Wujun Pan, Jingya Zhao, Yuming Xu

**Affiliations:** 1 Department of Neurology, Huaihe Hospital of Henan University, Kaifeng, 475000 China; 2 Department of Rehabilitation, Huaihe Hospital of Henan University, Kaifeng, 475000 China; 3 Department of Neurology, The First Affiliated Hospital of Zhengzhou University, No. 1 Jianshe East Road, Zhengzhou, 450052 China

**Keywords:** Parkinson disease, MALAT1, miR-124, Apoptosis

## Abstract

**Background:**

Parkinson disease (PD) is the most common movement disturbance characterized by the loss of dopaminergic (DA) neurons in midbrain. Metastasis-associated lung adenocarcinoma transcript 1 (MALAT1) is aberrantly expressed in neurons and is involved in the dendritic and synapse development. However, the role of MALAT1 and its underlying mechanism in PD remain to be defined.

**Methods:**

The expressions of MALAT1 and miR-124 were evaluated by qRT-PCR. N-methyl-4-phenyl-1,2,3,6-tetrahydropyridine (MPTP)-induced PD mice and SH-SY5Y cells subjected to N-methyl-4-phenylpyridinium (MPP^+^) were utilized to investigate the effect of MALAT1 on PD. TUNEL assay was performed to detect apoptosis of DA neurons in PD mice. Flow cytometry analysis was carried out to measure apoptosis of SH-SY5Y cells. Caspase3 activity and Cleaved Caspase3 expression were tested by caspase3 assay kit and western blot, respectively. TargetScan software and luciferase reporter assay were used to explore the relationship between MALAT1 and miR-124.

**Results:**

MALAT1 was up-regulated and miR-124 was down-regulated in MPTP-induced PD mice and MPP^+^-treated SH-SY5Y cells. MALAT1 knockdown attenuated MPTP-induced apoptosis of DA neurons in MPTP-induced PD mouse model. MALAT1 interacted with miR-124 to negatively regulate its expression. MALAT1 knockdown suppressed MPP^+^-induced apoptosis in SH-SY5Y cells, while miR-124 downregulation abrogated this effect. Moreover, MALAT1 knockdown improved miR-124 expression in MPTP/MPP^+^ induced models of PD.

**Conclusions:**

MALAT1 promotes the apoptosis by sponging miR-124 in mouse models of PD and in vitro model of PD, providing a potential theoretical foundation for the clinical application of MALAT1 against PD.

## Background

Parkinson’s disease (PD) is a progressive neurological disorder altering the movement abilities in adults, and has become the second most frequent chronic and systemic neurodegenerative disease, ranking only second to Alzheimer’s disease (AD) [1]. It has been estimated that approximately 8–18 new diagnosed PD cases will appear per 1,010,000 persons per year [2]. PD is characterized by the specific loss of dopaminergic (DA) neurons presented in the substantia nigra pars compacta (SNpc) along with widespread intracellular α-synuclein (α-syn) aggregates in several mammalian species, which triggered aberrant nerve signal that leads to movement coordination impairment and cognitive deficits [3]. Current treatments available for PD relieve the symptom instead of inhibiting the development of PD. Therefore, it is imperative to make consistent research efforts on strategies aimed at explaining the pathogenic mechanism at the molecular level and providing an effective therapy for PD.

Recently, non-coding RNAs (ncRNAs), especially long non-coding RNAs (lncRNAs) and microRNAs (miRNAs), have been suggested to play a crucial regulatory role in many cellular processes (e.g., brain development and differentiation) and pathological diseases (e.g., PD and AD) [4]. LncRNAs refer to a class of ncRNAs that are more than 200 nucleotides with no protein coding ability [5, 6]. It is well documented that lncRNAs are frequently up-regulated in brains and involved in many neurobiologic functions and neurodegenerative disorders [6–8]. For example, Liu et al. found that lncRNA Homeobox (HOX) transcript antisense RNA (HOTAIR) was highly expressed in the midbrain of PD mice induced by MPTP (N-methyl-4-phenyl-1,2,3,6- tetrahydropyridine) and SH-SY5Y cells pre-treated with MPP^+^ (N-methyl-4-phenylpyridinium), and promoted the onset of PD induced by MPTP through regulating LPPK2 (leucine-rich repeat kinase 2) [9]. A preliminary report pointed that the up-regulated lncRNA p21, SNHG1 and MALAT1 preceded the course of PD [8]. Metastasis-associated lung adenocarcinoma transcript 1 (MALAT1), also identified as a nuclear-enriched abundant transcript 2 (NEAT2), is a spliced lncRNA which is highly conversed in mammals and widely expressed in human tissues [10]. Amounting evidence indicated that abnormal expression of MALAT1 participated in tumorigenesis of various types of cancers [11–13]. Moreover, MALAT1 was demonstrated to be aberrantly expressed in neurons and modulate a series of genes associated with the dendritic and synapse development [14, 15]. However, the exact role of MALAT1 and its underlying mechanism in PD has not been clearly established.

miRNAs function as important regulators of gene expression in a variety of biological processes at the transcriptional or post-transcriptional level through targeting the 3′UTR of mRNAs [15]. Up to now, ample evidence suggests that dysregulated expression of miRNAs is implicated in DN biology and pathology of neurodegenerative diseases, including PD. For example, Zhou et al. revealed that miR-7 regulated neuroinflammation in the pathogenesis of PD by targeting Nod-like receptor protein 3 inflammasome [16]. Niu et al. found that miR-133b promoted axon outgrowth in DA neurons and ameliorated MPP^+^-induced axon degeneration by inhibiting RhoA [17]. Thome et al. noted that up-regulation of miR-155 promoted pro-inflammatory response to α-syn and aggravated α-syn-induced neurodegeneration in PD [18]. miR-124, a brain-enriched miRNA, has been demonstrated to play a neuroprotective role in some central nervous system diseases, such as autoimmune encephalomyelitis and stroke [19, 20]. Additionally, miR-124 has been illustrated to regulate apoptosis and autophagy in MPTP model of PD by targeting Bim [21].

In the present study, we investigated the role of MALAT1 in DA neurons apoptosis in PD and explored whether there existed an interaction between MALAT1 and miR-124 in PD.

## Methods

### Animals and treatment

The animal experiments were performed according to protocols from Research Committee and approved by the Institutional Animal Care and Use Committee, Huaihe Hospital of Henan University. Male C57BL/6 mice (10 week-old, weighing 20–25 g) were purchased from Chinese Academy of Medical Sciences Laboratory Animal Center (Beijing, China). All animals were housed in individual cages with standard pellet chow and water under a 12 h light/dark cycle. C57BL/6 mice were given an intraperitoneal injection of MPTP-HCl (30 mg/kg free base; Sigma, Saint Louis, Missouri, USA) per day for 4 consecutive days. An equivalent volume of 0.9% sterile saline was injected into mice as control. Six of randomly selected mice were sacrificed at 0 (instantly after the last MPTP injection), 1, 3, 5, and 7 days after the last MPTP injection. Mice were decapitated, and ventral midbrain containing the SNpc was resected and stored at −80 °C for subsequent analysis.

For exogenous delivery of MALAT1 in PD mice, a total of 48 C57BL/6 mice were randomly divided into 4 groups (n = 6/per group): NC group (0.9% sterile saline injection), MPTP group (intraperitoneal injection of MPTP-HCl), MPTP + sh-MALAT1 group (sh-MALAT1 infection along with intraperitoneal injection of MPTP-HCl) and MPTP + sh-control (sh-control infection along with intraperitoneal injection of MPTP-HCl). Mice recombinant lenti-shRNA vectors were established by inserting short hairpin RNA (shRNA) targeting MALAT1 (sh-MALAT1) or sh-control (GeneChem Co.Ltd, Shanghai, China) into linearized vector GV115 carrying green fluorescent protein (GFP) gene. Mice were placed into a stereotaxic frame (Stoelting, Wood Dale, IL, USA) under deep anesthesia using isoflurane in oxygen and nitrous oxide. The skull surface was exposed and a hole was drilled to aid positioning of the needle. Mice received bilateral injections of recombinant lenti-sh-control or lenti-sh-MALAT1 into the dorsal hippocampus (anterior −5.5; lateral +1.6; dorsoventral −7.5 from bregma) using a 5-μl Hamilton syringe with a 33-gauge tip needle, with 1 μl/side at a rate of 0.2 μl/min for 10 min. Two days after vector injection, administration of MPTP-HCl or sterile saline was performed as above.

### Cell culture and transfection

Human neuroblastoma cell line SH-SY5Y and human embryonic kidney cell line HEK293 were purchased from American Type Culture Collection (ATCC, Manassas, VA, USA). Cells were cultured in Dulbecco’s modified Eagle medium (DMEM) supplemented with 10% fetal bovine serum (FBS; Gibco, Grand Island, NY, USA), 100 U/ml penicillin and 100 μg/ml streptomycin in an atmosphere of 5% CO_2_ at 37 °C. SH-SY5Y cells were treated with 0.25, 0.5 or 1 mM MPP^+^ (Sigma, St. Louis, MO, USA) for 24 h to establish PD model in vitro.

siRNA against MALAT1 (si-MALAT1), siRNA control (si-control), pcDNA-MALAT1, pcDNA empty vector (vector), miR-124 mimics (miR-124), miRNA control (miR-control), miR-124 inhibitor (anti-miR-124) and negative control (anti-miR-control) were purchased from Ambion (Foster City, CA, USA). Cell transfection with oligonucleotides or vectors into SH-SY5Y cells was conducted by Lipofectamine 2000 (Invitrogen), followed by treatment of 1 mM MPP^+^ solution for 24 h.

### Quantitative real-time PCR

Total RNA from the resected midbrain and cultured SH-SY5Y cells was extracted using TRIzol Reagent (Invitrogen) as previously described [22]. The concentration and purity of the isolated RNA were detected using SMA 400 UV–vis (Merinton, Shanghai, China). Complementary DNA (cDNA) was synthesized from RNA samples using the reverse transcription system kit (Invitrogen). Real-time PCR for MALAT1 and miR-124 was conducted using the standard SYBR Green PCR kit protocol and TaqMan miRNA assays on a CFX96 real-time PCR System (Bio-Rad, Hercules, CA, USA), respectively. The results were normalized to GAPDH expression and calculated by the 2^−ΔΔCt^ method. The reaction conditions of quantitative PCR were as below: 95 °C for 10 min, 45 cycles of 20 s at 95 °C, 30 s at 60 °C, and 20 s at 72 °C. The specific primers used in the study were as follows: MALAT1 forward 5′-AAAGCAAGGTCTCCCCACAAG-3′, reverse: 5′-GGTCTGTGCTAGATCA AAAGGCA-3′; miR-124 forward 5′-CTAGTCTAGAGTCGCTGTTAT CTCATTGTCTG-3′; reverse, 5′-CGCGGATCCTCTGCTTCTGTCACAGAATC-3′; GAPDH forward 5′-TATGATGATATCAAGAGGGTAGT-3′, reverse 5′-TGTATCCAAACTCATTG TCATAC-3′.

### Western blot

Total protein samples from mouse midbrain or SH-SY5Y cells were extracted using NP-40 lysis buffer (Beyotime, Haimen, China). The concentration of the protein samples was quantified with the Bradford Protein Assay Kit (Beyotime). Equal amounts of protein sample (30–50 μg) were loaded on 10% sodium dodecyl sulfate polyacrylamide gel electrophoresis (SDS-PAGE), followed by transferred to polyvinylidenedifluoride (PVDF) membranes (Millipore, Billerica, MA, USA). After blocked with 5% non-fat milk for 1 h at room temperature, membrane was then incubated with rabbit anti-human antibodies Cleaved Caspase3 (1:500; Abcam, Cambridge, MA, USA) and β-actin (1:500; Cell Signaling Technology, Danvers, MA, USA) at 4 °C overnight. Subsequently, the corresponding horseradish peroxidase (HRP)-conjugated secondary antibody (1:5000; Cell Signaling Technology) was added for further incubation for 1 h at 37 °C. The blots were visualized with ECL chemiluminescent regents (Pierce, Rockford, IL, USA) and normalized to the internal control β-actin.

### Terminal deoxynucleotidyltransferase-biotin nick end-labeling (TUNEL) assay

Midbrain was fixed with 4% paraformaldehyde, dehydrated, paraffin-embedded and sectioned before TUNEL staining. Then sections were permeabilized with 20 μg/ml proteinase K (Solarbio Science & Technology Co., Ltd., Beijing, China) for 10 min. Apoptosis-specific nuclear DNA fragmentation in mouse midbrain was detected using the In Situ Cell Death Detection Kit (Roche Molecular Bioscience, Mannheim, Germany). The TUNEL-positive cell nucleus (stained brown) were visualized by an Axiovert 200 fluorescence microscope (Olympus, Tokyo, Japan), captured with a Photometrics SenSys cooled CCD camera (Roper Scientific, Tucson, AZ, USA). The percentage of apoptotic cells was determined by counting the TUNEL-positive cells and the total number of cells in 5 random high fields.

### Flow cytometry analysis

Cell apoptosis was assayed by Annexin V-FITC Apoptosis Detection Kit (Beyotime Institute of Biotechnology, Shanghai, China). Briefly, SH-SY5Y cells were collected, washed and resuspended in the annexin V binding buffer. Subsequently, cells were incubated with 5 μl Annexin V-FITC and stained with 5 μl PI at room temperature for 15 min. The flow cytometry data was analyzed using BD FACS Diva software V6.1.3 (BD Biosciences, San Jose, CA, USA).

### Caspase3 activity analysis

Caspase-3 activity was determined using the caspase3 assay kit (R&D Systems Inc., Minneapolis, MN, USA) according to the manufacturer’s instructions. Briefly, cells were lysed and centrifuged to obtain supernatants. The protein concentrations of each sample were quantified by the Bradford Protein Assay Kit (Beyotime). Then supernatants were mixed with buffer containing the substrate peptides for caspase-3 attached to p-nitroanilide (pNA) and incubated for 2 h at 37 °C. Absorbance at 405 nm was measured using a microplate reader (Bio-Rad Laboratories, Hercules, CA, USA).

### Luciferase reporter assay

The sequence of MALAT1 containing the predicted wild type of miR-124 was synthesized from Sangon (Shanghai, China) and cloned into the downstream of the Renilla luciferase gene of pGL3 vectors (Promega, Madison, WI, USA) to form the reporter vector pGL3-MALAT1-WT. To mutate the potential miR-124 binding sites in MALAT1 gene, a QuikChange Site-Directed Mutagenesis kit (Agilent Technologies, Palo Alto, CA, USA) was used for nucleotide-substitution mutation analysis following the manufacturer’s instructions. The constructed luciferase vectors were named as pGL3-MALAT1-MUT1/2. pRL-TK vector was used as an internal control. HEK293 cells were co-transfected with 50 ng recombinant luciferase vectors, 10 ng pRL-TK vectors and 50 nM miR-124 or miR-control by Lipofectamine 2000 (Invitrogen). After incubating the cells for 48 h, firefly and Renilla luciferase activities were measured using the Dual-Glo Luciferase assay system (Promega). The activity of renilla luciferase was normalized to that of firefly luciferase.

### Statistical analysis

Quantitative results were expressed as mean ± standard deviation (SD) (n = 3). Differences for three or more experimental groups were analyzed using one-way ANOVA tests. All data were analyzed using graph prism 5.0 software (GraphPad Prism, San Diego, CA). *P* value <0.05 was considered to be statistically significant.

## Results

### MALAT1 was up-regulated and miR-124 was down-regulated in MPTP-induced PD mouse model and in MPP^+^-intoxicated SH-SY5Y cells

**Fig. 1:**
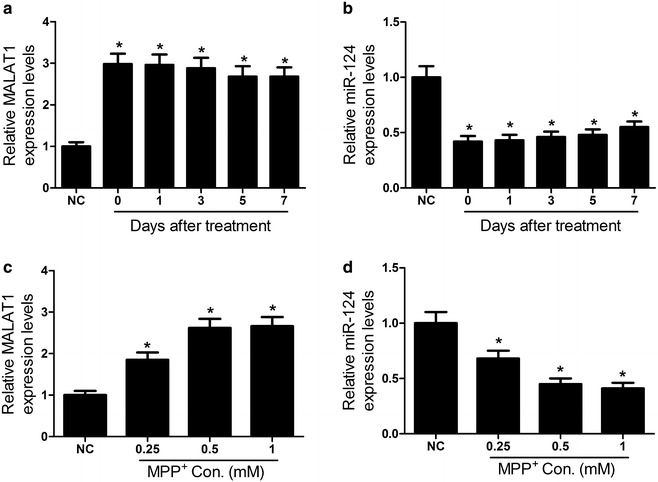
Expressions of MALAT1 and miR-124 in MPTP-induced PD mouse model and in MPP^+^-intoxicated SH-SY5Y cells. **a**, **b** The expressions of MALAT1 and miR-124 were examined by qRT-PCR in MPTP-induced PD mouse model. **c**, **d** The levels of MALAT1 and miR-124 were assessed by qRT-PCR in SH-SY5Y cells treated with 1 mM MPP^+^ for 24 h. *P < 0.05 vs. control group

Firstly, qRT-PCR was performed to determine the expressions of MALAT1 and miR-124 in the midbrain of MPTP-induced PD mouse model and MPP^+^-intoxicated SH-SY5Y cells. As shown in Fig. 1a, b, intraperitoneal injection of MPTP significantly increased MALAT1 expression and decreased miR-124 expression in mouse midbrains. Likely, MPP iodide also resulted in an obvious improvement of MALAT1 expression (Fig. 1c) and a remarkable reduction of miR-124 expression (Fig. 1d) in SH-SY5Y cells.

### MALAT1 knockdown attenuated apoptosis of DA neurons in MPTP-induced PD mouse model

**Fig. 2:**
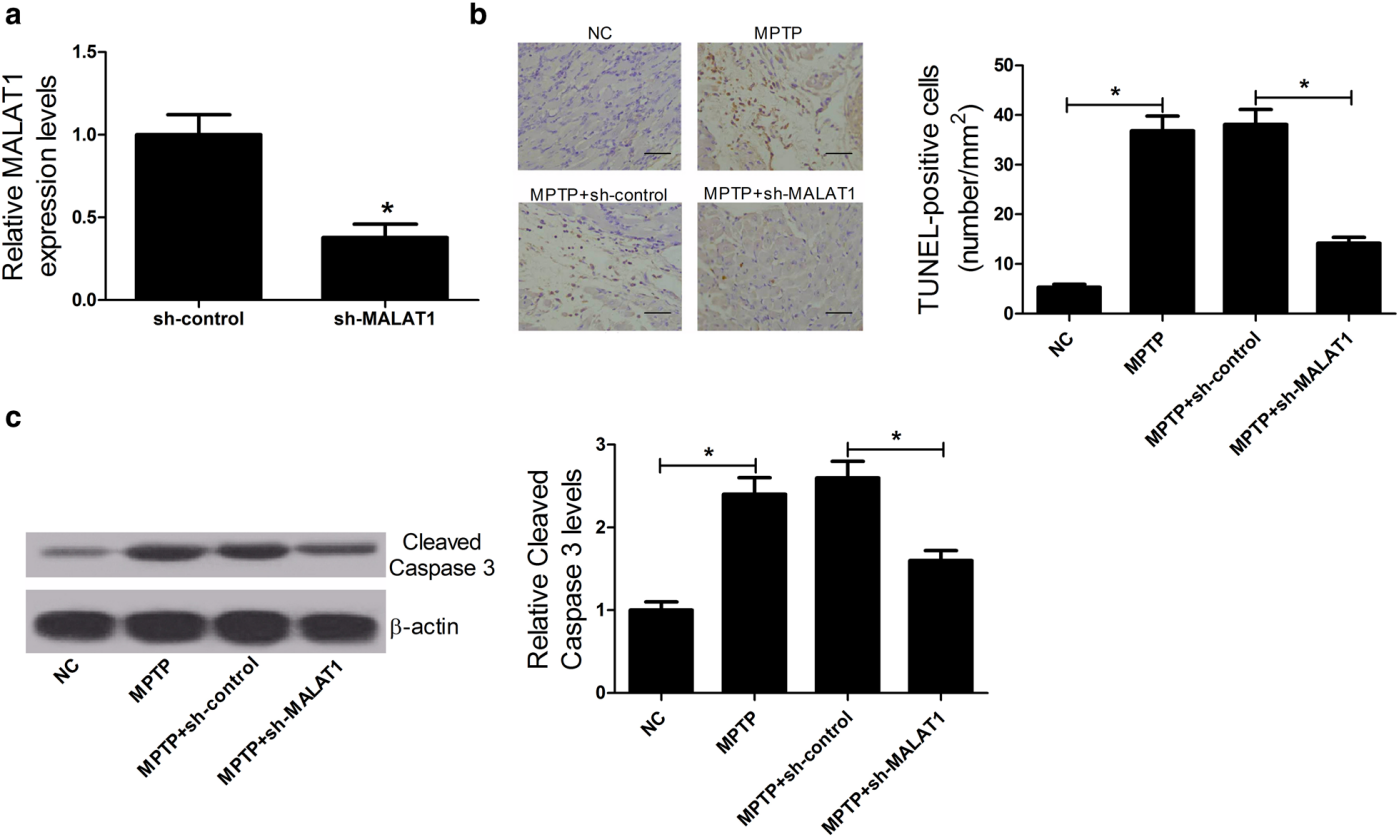
Effects of MALAT1 knockdown on apoptosis of DA neurons in MPTP-induced PD mouse model. Recombinant lentivirus vectors with sh-MALAT1 or sh-control were constructed and administered into the midbrain of mice. Two days post transfection, mice were treated with MPTP-HCl (30 mg/kg) by intraperitoneal injection for 4 days. Seven days after the last MPTP injection, mice were killed and ventral midbrain with SNpc was removed. **a** The expression of MALAT1 in sh-MALAT1- or sh-control-injected mice was detected by qRT-PCR. **b** TUNEL assay was carried out to analyze the apoptotic cells in treated mice (*scale bar* 50 μm). **c** Western blot was performed to assess the level of Cleaved Caspase3 in treated mice. β-actin was used as the internal control. *P < 0.05 vs. control group

Next, we investigated the effect of MALAT1 knockdown on apoptosis of DA neurons in MPTP-induced PD mouse model. Recombinant lentivirus vectors with sh-MALAT1 or sh-control were injected into the midbrain of mice. The downregulation of MALAT1 by sh-MALAT1 was confirmed by qRT-PCR (Fig. 2a). Two days after transfection, MPTP-HCl administration was performed. TUNEL assay showed that MPTP dramatically promoted apoptosis of DA neurons compared with negative control group, while MALAT1 knockdown conspicuously reduced MPTP-induced apoptosis of DA neurons with respect to sh-control transfection (Fig. 2b). Similarly, caspase3 activity assay revealed that the level of Cleaved Caspase3 in MPTP-treated mice was markedly elevated compared to NC group, whereas, lentivirus-mediated MALAT1 knockdown strikingly inhibited this effect (Fig. 2c). These findings indicated that MALAT1 knockdown alleviated apoptosis of DA neurons in MPTP-induced PD mouse model.

### MALAT1 negatively regulated miR-124 expression in HEK293 cells

**Fig. 3:**
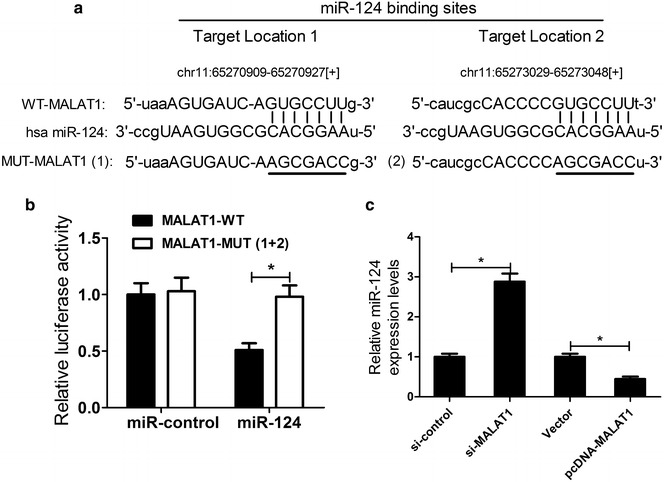
Interaction between MALAT1 and miR-124. **a** The putative recognition sequence of miR-124 in MALAT1 was predicted by Targetscan. **b** Luciferase activity was detected in HEK293 cells co-transfected with pGL3-MALAT1-WT or pGL3-MALAT1-MUT(1+2) and miR-124 or miR-control. **c** qRT-PCR was performed to evaluate miR-124 expression in SH-SY5Y cells transfected with si-MALAT1, pcDNA-MALAT1 or matched controls. *P < 0.05 vs. control group

A recent study suggested that lncRNAs could serve as a competing endogenous RNA (ceRNA) to modulate miRNA expression [23]. Thus, we predicted the potential miRNAs binding sequence for MALAT1 by using TargetScan software (Whitehead Institute, Cambridge, MA, USA). As displayed in Fig. 3a, there were two putative binding sites of miR-124 in MALAT1 transcripts. To further verify the interaction between MALAT1 and miR-124, we established luciferase reporter plasmids containing the wild-type or miR-124-binding site-mutant MALAT1 to co-transfect with miR-124 mimics or miR-control into HEK293 cells. The luciferase activities of pGL3-MALAT1-WT were significantly inhibited in miR-124-overexpressing HEK293 cells but miR-124 transfection exhibited no inhibitory effect on luciferase activities of pGL3-MALAT1-MUT (Fig. 3b). Furthermore, qRT-PCR was carried out to detect the expression of miR-124 in SH-SY5Y cells transfected with si-MALAT1 or pcDNA-MALAT1. The results showed that MALAT1 knockdown markedly improved miR-124 expression, while MALAT1 overexpression distinctly suppressed miR-124 expression in SH-SY5Y cells (Fig. 3c). Taken together, MALAT1 directly interacted with miR-124 and negatively regulated its expression.

### MALAT1 knockdown suppressed apoptosis of MPP^+^-intoxicated SH-SY5Y cells by sponging miR-124

**Fig. 4:**
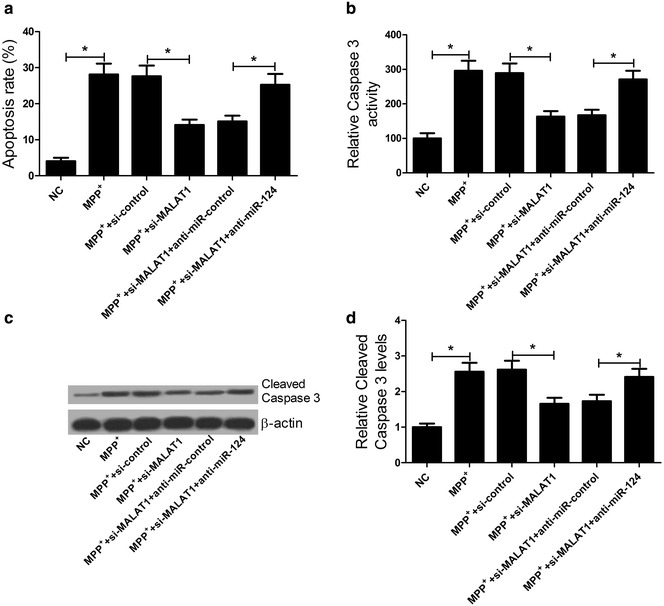
Effects of si-MALAT1 and anti-miR-124 on apoptosis of MPP^+^-intoxicated SH-SY5Y cells. SH-SY5Y cells were transfected with si-MATAL1 or in combination with anti-miR-124, followed by the treatment of 1 mM MPP^+^ solution for 24 h. **a** Flow cytometry analysis was performed to examine apoptosis in treated SH-SY5Y cells. **b** Caspase3 activity was determined by the caspase3 assay kit in treated SH-SY5Y cells. **c** Western blot was carried out to detect the level of Cleaved Caspase3 in treated SH-SY5Y cells. β-actin was used as the internal control. **d** Quantification analysis of Cleaved Caspase3. *P < 0.05 vs. control group

Based on the role of MALAT1 in DA neurons of PD model mouse induced by MPTP, we further explore the effect of MALAT1 knockdown on apoptosis in MPP^+^-intoxicated SH-SY5Y cells and its possible mechanism. As demonstrated by flow cytometry, MALAT1 knockdown significantly inhibited MPP^+^-induced apoptosis in SH-SY5Y cells, conversely, downregulation of miR-124 reversed this effect (Fig. 4a). Caspase3 kit assay indicated that the MALAT1 knockdown restrained increase of Caspase3 activity evoked by MPP iodide in SH-SY5Y cells, however, miR-124 inhibitor restored MALAT1 knockdown-suppressed Caspase3 activity (Fig. 4b). Similarly, western blot analysis showed that MALAT1 knockdown dramatically inhibited the expression of Cleaved Caspase3 level in MPP^+^-intoxicated SH-SY5Y cells, while cotransfection of si-MALAT1 and anti-miR-124 rescued this effect (Fig. 4c, d). Taken together, MALAT1 knockdown inhibited apoptosis by antagonizing miR-124 in MPP^+^-intoxicated SH-SY5Y cells.

### MALAT1 knockdown improved miR-124 expression in MPTP-induced PD mouse model and in MPP^+^-intoxicated SH-SY5Y cells

**Fig. 5:**
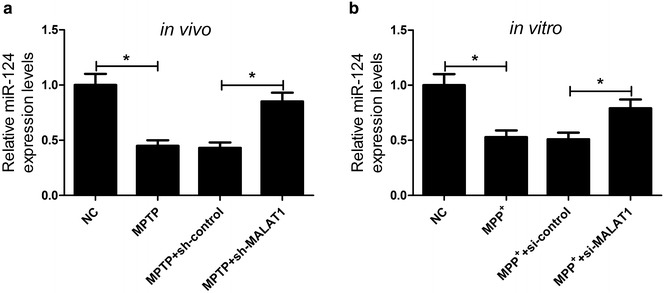
Effects of MALAT1 knockdown on expression of miR-124 in MPTP-induced PD mouse model and in MPP^+^-intoxicated SH-SY5Y cells. The expression of miR-124 was examined by qRT-PCR in MPTP-induced PD mouse model transfected with sh-MALAT1 or sh-control (**a**) and in MPP^+^-intoxicated SH-SY5Y cells transfected with si-MALAT1 or si-control (**b**). *P < 0.05 vs. control group

qRT-PCR was performed to further confirm the effect of MALAT1 knockdown on the expression of miR-124 in in vivo and in vitro model of PD. The results suggested that sh-MALAT1 transfection significantly relieved down-regulation of miR-124 induced by MPTP in mice (Fig. 5a). Consistently, transfection of si-MALAT1 dramatically abated down-regulation of miR-124 induced by MPP^+^ in SH-SY5Y cells (Fig. 5b), suggesting that MALAT1 could negatively regulate miR-124 expression in MPTP-induced PD mouse model and MPP^+^-intoxicated SH-SY5Y cells.

## Discussion

PD is a chronic, neurodegenerative disorder characterized by some pathological hallmarks such as the progressive death of DA neurons containing SNpc which are more susceptible to degeneration and the presence of intraneuronal cytoplasmic aggregates of α-Syn in Lewy bodies [24]. Despite many years of substantial researches, the etiology of PD is still not yet explained. Increasing evidence has demonstrated that lncRNAs are involved in gene regulation of synaptic plasticity, cognitive function and memory [25]. Besides, lncRNAs may function as the exquisite biomarkers for PD and other neurodegenerative diseases [26]. MALAT1 is highly expressed in neurons and MALAT1 acts as a regulator of gene expression involved in synapse formation and/or maintenance [14, 15]. In the present study, we established animal models of PD and in vitro model of PD by utilizing neurotoxin MPTP and MPP^+^ (the active metabolite of MPTP) to investigate the role of MALAT1 in PD [27, 28]. We found that MALAT1 expression was significantly elevated in MPTP/MPP^+^ models. Loss-of-function analysis showed that MALAT1 knockdown inhibited MPTP-induced apoptosis of DA neurons in PD mice, suggesting that MALAT1 contributed to PD neurodegeneration. In agreement with our results, a previous study indicated that MALAT1 was up-regulated in midbrain tissues of MPTP-induced PD mice and MPP^+^-treated SH-SY5Y cells, and MALAT1 may be a promising target molecular involving in the pathogenesis of PD [27].

Recently, it has been stated that lncRNAs serve as miRNA sponges or antagomirs to competitively regulate the expressions and activities of specific miRNAs [29]. MALAT1 was reported to be an endogenous regulator of breast cancer progression by down-regulating miR-124 and activating the CDK4/E2F1 signaling pathway [23]. MALAT1 could modulate GRB2 expression via competing miR-124 to promote high-risk human papillomavirus (HR-HPV)-positive cervical cancer cell growth and invasion [30]. Here, our study further investigated whether MALAT1 could regulate apoptosis of DA neurons in PD by sponging miR-124. Convincing evidence shows that miR-124 alteration is implicated in many CNS diseases, such as medulloblastoma, injured hypoglossal motor neurons and cerebral ischemic stroke [20, 31, 32]. Besides, miR-124 was reported to be down-regulated in neurons from MPTP-induced PD [33]. Our study confirmed the reduced expression of miR-124 in MPTP/MPP^+^ models. Additionally, TargetScan software prediction and luciferase reporter system demonstrated that MALAT1 could directly bind to miR-124 and negatively regulate its expression. Further studies revealed miR-124 inhibitor abolished inhibitory effect on apoptosis triggered by MALAT1 knockdown in MPP^+^-treated SH-SY5Y cells. Also, MALAT1 knockdown conspicuously reversed MPTP/MPP^+^ induced miR-124 expression suppression in in vivo and in vitro model of PD. These results suggested that MALAT1 knockdown inhibited apoptosis of DA neurons in MPTP-induced PD mice, as well as suppressed apoptosis of MPP^+^-treated SH-SY5Y cells by sponging miR-124.

## Conclusions

In summary, our study demonstrated that MALAT1 was up-regulated and miR-124 was down-regulated in MPTP/MPP^+^ models. MALAT1 contributed to apoptosis of DA neurons by sponging miR-124 in mouse models of PD and in vitro model of PD, providing a potential theoretical foundation for the clinical application of MALAT1 against PD.

## Abbreviations

PD: Parkinson disease
DA: dopaminergic
MALAT1: metastasis-associated lung adenocarcinoma transcript 1
AD: Alzheimer’s disease
SNpc: substantia nigra pars compacta
α-syn: α-synuclein
ncRNAs: non-coding RNAs
lncRNAs: long non-coding RNAs
miRNAs: microRNAs
HOX: homeobox
NEAT2: nuclear-enriched abundant transcript 2
shRNA: short hairpin RNA
GFP: green fluorescent protein
cDNA: complementary DNA
SDS-PAGE: sodium dodecyl sulfate polyacrylamide gel electrophoresis
PVDF: polyvinylidene difluoride
pNA: p-nitroanilide
HR-HPV: high-risk human papillomavirus
